# Phenotyping lipid profiles in type 2 diabetes: Risk association and outcomes from the Cardiovascular Health Study

**DOI:** 10.1016/j.ajpc.2024.100725

**Published:** 2024-08-26

**Authors:** David Bleich, Mary L. Biggs, Julius M. Gardin, Mary Lyles, David S. Siscovick, Kenneth J. Mukamal

**Affiliations:** a Division of Endocrinology, Diabetes, & Metabolism, Rutgers New Jersey Medical School, 185 South Orange Avenue, MSB I-588, Newark, NJ 07103, United States; b University of Washington School of Medicine, Seattle, WA, United States; c Wake Forest School of Medicine, Winston-Salem, NC, United States; d The New York Academy of Medicine, New York, NY, United States; e Harvard Medical School, Boston, MA, United States

**Keywords:** Type 2 diabetes, Cardiovascular risk, Lipid phenotyping

## Abstract

**Aims:**

To determine whether discrete lipid profiles (refer to as lipid phenotyping) can be used to stratify cardiovascular risk in individuals with type 2 diabetes.

**Methods and results:**

Cardiovascular Health Study participants with diabetes and fasting lipid profiles at baseline (*n* = 866) were categorized separately by level of LDL cholesterol and HDL-C/Triglyceride (Tg) profiles (low Tg/high HDL-C; high Tg/low HDL-C; high Tg only or low HDL-C only). We performed Cox multivariate regression analysis to assess the risk of CVD mortality, incident myocardial infarction (MI), heart failure (HF), stroke, and composite MACE (MI, HF, stroke, and CVD mortality) associated with each lipid category. We also calculated risk estimates for MACE using lipid measures as continuous variables. In the fully adjusted model, the high triglyceride plus low HDL-C cholesterol phenotype demonstrated risk that was at least as high as the highest LDL-C sub-group phenotype for CVD mortality (Hazard ratio {HR} 1.58 vs 1.48), MI (HR 1.53 vs 1.58), HF (HR 1.47 vs 1.20), stroke (HR 2.02 vs 1.43), and MACE (HR 1.58 vs 1.38). When modeled continuously, the HR per SD for MACE was 1.12 (*p* = 0.03) for LDL-C and 1.19–1.20 (*p* < 0.001) for triglycerides or remnant cholesterol.

**Conclusions:**

Participants with the high triglyceride/low HDL-C phenotype had equivalent or higher CVD risk than those with the high LDL-C phenotype. Further studies are necessary to determine whether lipid phenotyping accounts for the substantial CVD risk not explained by LDL cholesterol among individuals with type 2 diabetes.

**Figure:**
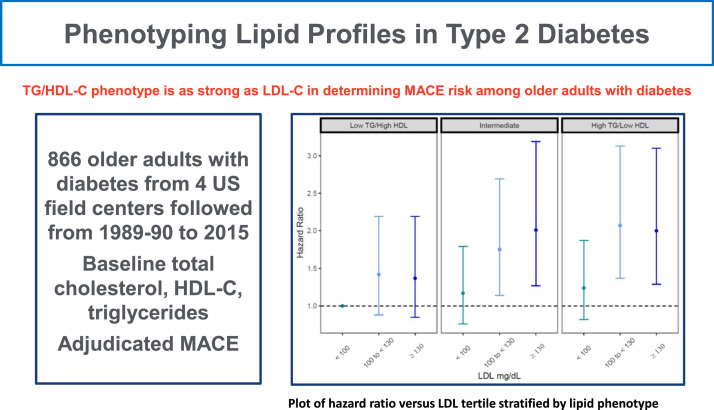
Central Illustration.

## 1 Introduction

In major randomized placebo-controlled, double-blinded CVD studies, LDL lowering has conferred an approximately 30 percent risk reduction for major adverse cardiovascular events (MACE) [1]. Typically, these studies are enriched with type 2 diabetic (T2DM) subjects, who may account for greater than 50 percent of study participants. However, there is still approximately 70 percent remaining CVD risk reduction potential that is left unaddressed. The present treatment strategies focused on LDL reduction (e.g., statin therapy) are not fundamentally flawed, but in 2024 we may have reached the limit of maximizing CVD risk reduction in those individuals with T2DM and dyslipidemia even with the addition of the newer proprotein convertase subtilisin/kexin type 9 (PCSK-9) inhibitors. Furthermore, we have concluded that raising high-density lipoprotein (HDL) cholesterol with niacin or HDL-targeted drugs does not yield additional cardiovascular risk reduction [2,3]. So too, it might be the case that lowering triglyceride and VLDL cholesterol with fibrate drugs does not decrease CVD risk in individuals with type 2 diabetes, as recently shown [4]. Therefore, newer approaches to navigate CVD risk in patients with T2DM are warranted. This observation is further validated in the recently published AHA Presidential Advisory on Life's Essential 8 where revised lipid guidelines now include non-HDL cholesterol, an acknowledgement that LDL cholesterol is not the whole story [5].

Subjects with T2DM have diverse phenotypic lipid profiles that are readily discernible in any diabetes clinic. These phenotypes can be categorized as: 1. High LDL-C (hLDL) only; 2. High triglyceride (hTg) plus low HDL-C (loHDL); and 3. Intermediate phenotypes with high triglyceride only or low HDL-C only. Each of these phenotypes is informed by genetic and molecular underpinnings. For example, the hLDL phenotype is due in part to increased LDL receptor (LDLr) recycling and degradation with low LDLr expression on the hepatocyte cell surface [6]. Newer therapy with the PCSK-9 inhibitor class inhibits LDL receptor recycling, preserving LDL receptors on the hepatocyte cell surface leading to dramatic lowering of LDL cholesterol.

In distinction, the hTg/loHDL phenotype occurs with insulin resistance in the liver that leads to up-regulation of Apo CIII and subsequent inhibition of lipoprotein lipase (LPL) [7,8]. Secondary effects of LPL inhibition result in high serum triglyceride levels with concomitant VLDL degradation to small dense LDL, a highly atherogenic particle. Reverse cholesterol transport is also impaired when HDL is converted into small dense particles [9]. These small dense HDL particles are broken down in the liver and APO AI, the major apolipoprotein of HDL, exits the circulation through the urine [10]. Moreover, the hTg/loHDL phenotype leads to increased fatty acid flux through the lipid scavenging receptor CD36 [11]. When combined with impaired reverse cholesterol transport, this dual defect has deleterious consequences on the vascular endothelium [12]. Genetic studies on individuals with loss of function mutation in the APOC3 gene reveal significant low serum triglyceride levels and decreased CVD risk [13].

These different lipid phenotypes, commonly found in patients with T2DM, reflect different molecular mechanisms of disease. Recognition of these differences was described by John Brunzell and colleagues in 1992 as familial combined hyperlipidemia with reduced lipoprotein lipase activity [14].

We assessed the association of lipid phenotypes on CVD risk in participants with T2DM in the Cardiovascular Health Study (CHS), which has followed a large cohort of older individuals for more than 30 years [15]. The objective of this study was to determine whether lipid phenotyping in subjects with T2DM provides additional CVD risk stratification beyond simple measurement of LDL cholesterol. We postulated that the hTg/loHDL phenotype would confer higher CVD risk in diabetic subjects compared to those with isolated LDL elevation or an intermediate phenotype.

## 2 Methods

### 2.1 Study population and design

The CHS is a prospective, population-based, longitudinal study of coronary heart disease and stroke in adults aged 65 years and older [15]. Study subjects were initially recruited between 1989 and 1990 from Medicare eligible registries at four different field centers with a target goal of 1250 subjects per study site and ultimate enrollment of 5201 individuals in total. A second recruitment phase occurred 3 years later when an additional 687 Black subjects were enrolled. Full details of study enrollment and baseline data collection have been previously reported [16].

All CHS participants provided written informed consent, and the CHS protocol was reviewed and approved by Institutional Review Boards at all participating institutions. All data used in this analysis, except for incident CVD and mortality events, were obtained at the baseline CHS examination (i.e. 1989–1990 or 1992–1993). Information on sociodemographic, lifestyle, and clinical risk factors was obtained via questionnaires administered by trained interviewers.

### 2.2 Lipid and diabetes assessment

Total cholesterol, high-density lipoprotein (HDL) cholesterol, triglyceride and glucose were directly measured in blood samples obtained from participants after an overnight 8–12-hour fast [17]. LDL cholesterol was calculated using the Friedewald equation [18] and secondarily using the updated NIH equation [19]. We also calculated non-HDL cholesterol (i.e., total cholesterol minus HDL-C) and remnant cholesterol (i.e., total cholesterol minus both HDL-C and LDL-C). Medication use among participants was assessed by clinic staff using a medication inventory [20]. We identified patients with type 2 diabetes based on fasting glucose ≥ 126 mg/dl, non-fasting glucose ≥ 200 mg/dL, or use of oral hypoglycemic agents or insulin injections.

### 2.3 Cardiovascular event ascertainment

Surveillance, ascertainment and adjudication of CVD and mortality has been described previously [21]. In brief, participants were asked about possible cardiovascular events and all hospitalizations. Field center staff then obtained discharge summaries for all hospital stays. For all adjudicated incident cardiovascular events (including myocardial infarction, stroke, and heart failure), relevant diagnostic information was also collected. The CHS Cardiovascular/Cerebrovascular Committees then reviewed and adjudicated all events blinded to data collection at CHS visits.

### 2.4 Statistical analysis

We used clinically relevant, predetermined cut points for assigning study subjects into lipid phenotype groups. For LDL cholesterol, we divided individuals into three groups (high, intermediate, and low risk), those with LDL-C ≥ 130 mg/dL, >100 to <130 mg/dL, and <100 mg/dL, respectively. For fasting triglyceride level, we defined >160 mg/dl as high. For HDL cholesterol, we defined ≤ 50 mg/dL for women and ≤ 40 mg/dL for men as low. In some instances, study participants had high triglyceride with normal HDL-C or normal triglyceride with low HDL-C; we classified them as “intermediate ".

We calculated mean and standard deviation (SD) for continuous variables and proportions for categorical variables to describe the sociodemographic, lifestyle, and clinical characteristics across categories of LDL and of TG/HDL-C. To adjust for potentially confounding variables that might be related to lipid levels and/or risk of cardiovascular disease, we used Cox proportional hazards regression to estimate adjusted hazard ratios (as estimates of relative risk) for several related binary endpoints. These include incident myocardial infarction, incident heart failure, incident stroke, CVD mortality, all-cause mortality, and composite MACE (a composite of MI, HF, stroke, and CVD mortality). Time at risk was calculated as the interval in days from the baseline examination to the earliest of the following: date of event, date of death, date of last follow-up, or administrative censoring date of June 2015.

To explore the importance of sets of confounders, we fit three sequential models. Model 1 adjusted for age, sex, race (black, non-black), and field center as core demographic variables. Model 2 additionally adjusted for cardiovascular risk factors, including smoking status (never, former, current), BMI, systolic blood pressure, diastolic blood pressure, hypertension medication use, lipid-lowering medication use, insulin use, and oral hypoglycemic medication use. Model 3 additionally adjusted for the other lipid phenotype (i.e., LDL-C vs TG/HDL-C). We tested equality of the LDL-C and TG/HDL-C HR estimates in Model 3 using a Wald test (i.e., to compare the strengths of associations of LDL-C and TG/HDL-C in relation to MACE). We evaluated the validity of the proportional hazards’ assumption using log-log plots and Schoenfeld residuals and found no evidence of meaningful non-proportionality.

In secondary analyses, we evaluated the potential interaction (i.e., positive or negative synergy) between LDL-C and TG/HDL-C with MACE by fitting a model with cross-product terms (i.e., each combination of LDL-C categories and TG/HDL-C categories). We then estimate the hazard ratios across the 9 joint LDL-C and TG/HDL-C categories, using the lowest joint category as the reference.

## 3 Results

### 3.1 Lipid phenotypes

We identified a total of 925 participants with diabetes at baseline. Of these, *n* = 45 were missing LDL cholesterol values, and 14 had not fasted for at least 8 h prior to blood draw, leaving *n* = 866 for the analysis. When sub-grouped by LDL cholesterol, 25.5% were categorized as low risk <100 mg/dL (*n* = 221); 32.2% were intermediate risk ≥ 100 to <130 mg/dL (*n* = 279); and 42.3% were high risk ≥ 130 mg/dl (*n* = 366). Mean LDL cholesterol in each group (± SD) was 80.2 ± 15.5 mg/dL, 115.2 ± 8.7 mg/dL, and 160.8 ± 26.3 mg/dL, respectively. LDL cholesterol estimated by the NIH method was nearly perfectly correlated with the Friedewald method (*r* = 0.998).

When participants were stratified into triglyceride and HDL-C phenotypes, 38.3% had low triglyceride plus high HDL-C (loTG/hHDL) (*n* = 332), 28.5% had high triglyceride plus low HDL-C (hTg/loHDL; *n* = 247) and 33.1% were intermediate (*n* = 287). These intermediate study subjects had either high triglyceride plus “normal HDL” or “normal” triglyceride plus low HDL, so they did not fit into one of our a priori phenotypic groups.

In Table 1, we stratified CHS participants by Tg/HDL phenotypes. Strata did not differ meaningfully in age, blood pressure, BMI, LDL cholesterol, fasting glucose, oral hypoglycemic agents or insulin use, field center, education status, alcoholic beverages consumed per week, physical activity, or cigarette smoking status. There were fewer black participants with the hTG/lHDL phenotype. Comparable values by LDL-C are shown in Supplemental Table 1.

**Table 1:** Characteristic of CHS participants with diabetes at baseline, by TG/HDL phenotype.

	Low TG/High HDL* (*n* = 332)	Intermediate* (*n* = 287)	High TG/Low HDL* (*n* = 247)
Age (years)	73.1 ± 5.9	72.8 ± 5.0	72.9 ± 5.4
Male	52.4%	48.8%	46.6%
Black race	31.3%	24.4%	13.0%
Field center			
Bowman Gray	25.3%	26.1%	24.7%
Davis	25.0%	18.8%	17.4%
Hopkins	19.6%	26.1%	29.1%
Pittsburgh	30.1%	28.9%	28.7%
Educational attainment			
<HS	37.7%	35.2%	37.2%
HS	25.9%	29.3%	26.3%
>HS	36.4%	35.5%	36.4%
Body mass index (kg/m2)	27.7 ± 5.3	28.9 ± 5.0	29.4 ± 4.3
Diastolic BP (mmHg)	143.8 ± 21.9	138.5 ± 21.9	138.0 ± 20.3
Systolic BP (mmHg)	71.9 ± 11.9	70.3 ± 12.0	69.6 ± 11.7
LDL (mg/dl)	124.2 ± 36.8	129.3 ± 37.9	123.0 ± 40.2
HDL (mg/dl)	57.3 ± 12.2	45.2 ± 10.5	38.0 ± 6.3
Triglyceride (mg/dl)	105.2 ± 27.8	154.9 ± 55.7	235.7 ± 60.6
Fasting glucose (mg/dl)	165.4 ± 60.0	169.1 ± 54.1	178.3 ± 66.6
Any anti-hypertensive medication	59.3%	63.1%	70.4%
Any lipid-lowering medication	8.1%	6.6%	8.1%
Oral hypoglycemic agents	41.3%	41.5%	37.7%
Insulins	15.4%	12.5%	15.4%
CHD	23.8%	27.2%	34.0%
CHF	7.2%	7.3%	12.1%
Stroke	7.2%	7.3%	6.1%
No. alcoholic beverages/wk	2.1 ± 5.8	1.2 ± 4.0	1.0 ± 4.0
Physical activity (kcal/wk)	1652.7 ± 2317.5	1494.3 ± 1970.4	1273.4 ± 1654.7
Smoking status			
Never smoked	47.0%	47.4%	44.1%
Former smoker	45.5%	40.4%	45.3%
Current smoker	7.5%	12.2%	10.5%

High TG defined as TG > 160 mg/dL; low HDL defined as ≤40 mg/dL (men) or ≤50 mg/dL (women).

Figures shown are mean ± SD for continuous measures and percentages for categorical measures.

The crude incidence of cardiovascular events at baseline in diabetic CHS participants stratified by LDL-C and hTg/lHDL phenotypes is shown in Supplemental Tables 2 and 3, respectively. As anticipated, CVD events were substantially higher in the hTg/lHDL and intermediate phenotypes (either hTG or lHDL only) compared to the lTG/hHDL group. In contrast, CVD events were marginally higher between the lowest and highest tertiles of LDL cholesterol. This result has been previous seen in other epidemiologic studies where elevated non-fasting triglyceride level was strongly associated with CVD events [22].

### 3.2 Association of continuous lipid measures and MACE

As shown in Table 2, a 1-SD increase in triglycerides, non-HDL cholesterol, and remanent cholesterol resulted in equivalent, highly statistically significant hazard ratios for MACE of ∼ 1.20. In contrast, LDL-C and HDL-C were numerically more weakly associated with risk, regardless of which LDL-C estimator was used.

**Table 2:** Association of continuous lipid measures and MACE.

Measure	HR per SD (95% CI)	p-value
LDL-C (Friedewald)	1.12 (1.01–1.23)	0.03
LDL-C (NIH Equation)	1.13 (1.02–1.24)	0.02
HDL-C	0.89 (0.80–0.99)	0.04
Log Triglyceride	1.19 (1.08–1.32)	<0.001
Log Non-HDL-C	1.19 (1.08–1.31)	0.001
Log Remnant Cholesterol	1.20 (1.08–1.32)	<0.001

Model adjusts for age, sex, race (Black, non-Black), field center, smoking status (never, former, current), BMI, systolic blood pressure, diastolic blood pressure, hypertension medication use, lipid-lowering medication use, insulin use, oral hypoglycemic medication use.. (SD) = standard deviation.

### 3.3 Adjusted models of lipid phenotypes and outcomes in CHS participants with diabetes

Regardless of the model, the hTg/loHDL phenotype demonstrated the highest hazard ratios for all outcomes except all-cause mortality and incident MI (Table 3). Model 3 controls for the greatest number of confounders and includes mutual adjustment of LDL-C and TG/HDL-C to assess their independent associations. When comparing CVD mortality between the hLDL phenotype and the hTg/loHDL phenotype, the hazard ratios were 1.45 versus 1.58. Further numeric comparison of these two high-risk groups showed approximate equal risk for incident MI (1.58 versus 1.53), greater risk for HF in the hTg/loHDL group (1.47 versus 1.20), greater risk for stroke in the hTg/loHDL group (2.02 versus 1.43), and greater risk for MACE in the hTg/loHDL group (1.58 versus 1.38). Although every comparison showed higher risk for the hTg/loHDL group, only the differences in stroke risk were statistically significantly stronger for hTg/loHDL than LDL-C (*p* = 0.03).

**Table 3:** Associations of lipid phenotypes with outcomes among CHS participants with diabetes.

	**Model 1**	**Model 2**	**Model 3**
**All-cause mortality**	**HR (95% CI); p-value**	**HR (95% CI); p-value**	**HR (95% CI); p-value**
LDL-C			
<100	1.00	1.00	1.00
>=100-<130	0.95 (0.79–1.14); *p* = 0.55	0.95 (0.79–1.15); *p* = 0.62	0.95 (0.79–1.15); *p* = 0.63
>=130	1.04 (0.87–1.24); *p* = 0.68	1.08 (0.90–1.30); *p* = 0.40	1.09 (0.91–1.31); *p* = 0.35
TG/HDL-C			
Low TG/High HDL-C	1.00	1.00	1.00
Intermediate	1.15 (0.98–1.35); *p* = 0.09	1.21 (1.02–1.43); *p* = 0.03	1.21 (1.02–1.43); *p* = 0.03
High TG/Low HDL-C	1.18 (0.99–1.40); *p* = 0.06	1.19 (1.00–1.43); *p* = 0.05	1.20 (1.00–1.43); *p* = 0.05
Test LDL-C=TG/HDL-C			*p* = 0.13
**CVD mortality**	**HR (95% CI); p-value**	**HR (95% CI); p-value**	**HR (95% CI); p-value**
LDL-C			
<100	1.00	1.00	1.00
>=100-<130	1.17 (0.88–1.54); *p* = 0.28	1.17 (0.88–1.55); *p* = 0.28	1.20 (0.90–1.60); *p* = 0.21
>=130	1.34 (1.03–1.75); *p* = 0.03	1.44 (1.10–1.89); *p* = 0.01	1.48 (1.13–1.95); *p* = 0.01
TG/HDL-C			
Low TG/High HDL-C	1.00	1.00	1.00
Intermediate	1.26 (0.99–1.60); *p* = 0.06	1.38 (1.08–1.77); *p* = 0.01	1.38 (1.08–1.77); *p* = 0.01
High TG/Low HDL-C	1.45 (1.13–1.86); *p* < 0.001	1.55 (1.19–2.01); *p* < 0.001	1.58 (1.22–2.06); *p* < 0.001
Test LDL-C=TG/HDL-C			*p* = 0.54
**Incident MI**	**HR (95% CI); p-value**	**HR (95% CI); p-value**	**HR (95% CI); p-value**
LDL-C			
<100	1.00	1.00	1.00
>=100-<130	1.30 (0.86–1.97); *p* = 0.22	1.32 (0.86–2.02); *p* = 0.20	1.35 (0.88–2.07); *p* = 0.17
>=130	1.48 (0.99–2.22); *p* = 0.06	1.54 (1.02–2.33); *p* = 0.04	1.58 (1.04–2.40); *p* = 0.03
TG/HDL-C			
Low TG/High HDL-C	1.00	1.00	1.00
Intermediate	1.26 (0.89–1.79); *p* = 0.19	1.37 (0.95–1.98); *p* = 0.09	1.37 (0.95–1.97); *p* = 0.09
High TG/Low HDL-C	1.41 (0.97–2.04); *p* = 0.07	1.48 (1.00–2.17); *p* = 0.05	1.53 (1.04–2.26); *p* = 0.03
Test LDL-C=TG/HDL-C			*p* = 0.96
**Incident HF**	**HR (95% CI); p-value**	**HR (95% CI); p-value**	**HR (95% CI); p-value**
LDL-C			
<100	1.00	1.00	1.00
>=100-<130	1.25 (0.94–1.66); *p* = 0.12	1.25 (0.94–1.67); *p* = 0.13	1.29 (0.96–1.73); *p* = 0.09
>=130	1.11 (0.84–1.47); *p* = 0.47	1.18 (0.88–1.57); *p* = 0.27	1.20 (0.90–1.60); *p* = 0.21
TG/HDL-C			
Low TG/High HDL-C	1.00	1.00	1.00
Intermediate	1.29 (1.00–1.65); *p* = 0.05	1.33 (1.03–1.72); *p* = 0.03	1.32 (1.02–1.72); *p* = 0.03
High TG/Low HDL-C	1.46 (1.12–1.90); *p* = 0.01	1.43 (1.09–1.88); *p* = 0.01	1.47 (1.12–1.93); *p* = 0.01
Test LDL-C=TG/HDL-C			*p* = 0.53
**Incident Stroke**	**HR (95% CI); p-value**	**HR (95% CI); p-value**	**HR (95% CI); p-value**
LDL-C-C			
<100	1.00	1.00	1.00
>=100-<130	0.99 (0.65–1.49); *p* = 0.95	1.06 (0.69–1.61); *p* = 0.80	1.07 (0.70–1.63); *p* = 0.76
>=130	1.38 (0.95–2.02); *p* = 0.10	1.40 (0.95–2.07); *p* = 0.09	1.43 (0.97–2.12); *p* = 0.07
TG/HDL-C			
Low TG/High HDL-C	1.00	1.00	1.00
Intermediate	1.84 (1.29–2.64); *p* < 0.001	2.19 (1.51–3.16); *p* < 0.001	2.16 (1.50–3.12); *p* < 0.001
High TG/Low HDL-C	1.73 (1.18–2.53); *p* < 0.001	1.99 (1.34–2.94); *p* < 0.001	2.02 (1.37–3.00); *p* < 0.001
Test LDL-C=TG/HDL-C			*p* = 0.03
**MI, Stroke, HF, CVD mortality**	**HR (95% CI); p-value**	**HR (95% CI); p-value**	**HR (95% CI); p-value**
LDL-C-C			
<100	1.00	1.00	1.00
>=100-<130	1.21 (0.94–1.57); *p* = 0.14	1.24 (0.96–1.61); *p* = 0.10	1.27 (0.97–1.65); *p* = 0.08
>=130	1.28 (1.00–1.64); *p* = 0.05	1.36 (1.06–1.76); *p* = 0.02	1.38 (1.07–1.78); *p* = 0.01
TG/HDL-C			
Low TG/High HDL-C	1.00	1.00	1.00
Intermediate	1.53 (1.23–1.90); *p* < 0.001	1.61 (1.28–2.01); *p* < 0.001	1.60 (1.28–2.00); *p* < 0.001
High TG/Low HDL-C	1.47 (1.16–1.86); *p* < 0.001	1.55 (1.21–1.98); *p* < 0.001	1.58 (1.24–2.03); *p* < 0.001
Test LDL-C=TG/HDL-C			*p* = 0.24

*High TG defined as TG > 160 mg/dL; low HDL defined as ≤40 mg/dL (men) or ≤50 mg/dL (women). Model 1 adjusts for age, sex, race (Black, non-Black), and field center. Model 2 adjusts for Model 1 covariates plus smoking status never, former, current), BMI, systolic blood pressure, diastolic blood pressure, hypertension medication use, lipid-lowering medication use, insulin use, oral hypoglycemic medication use.

Model 3 adjusts for Model 2 covariates plus the other lipid phenotype.

**Fig. 1:**
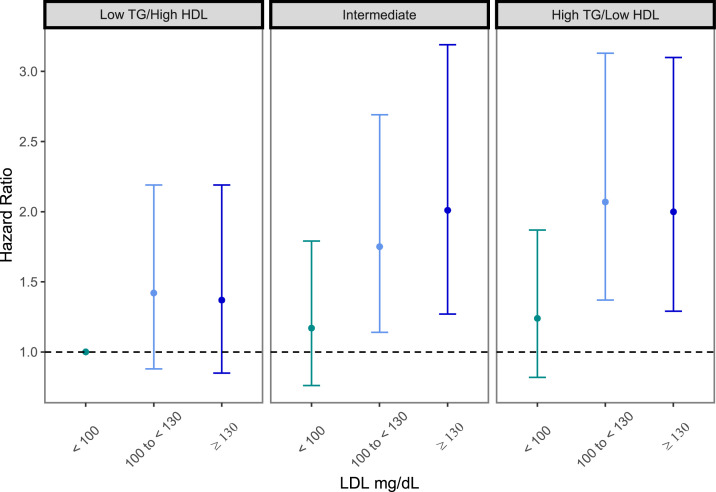
Hazard ratios and 95% confidence intervals for MACE across joint LDL and TG/HDL categories. Model is adjusted for age, sex, race (Black, non-Black), field center, smoking status (never, former, current), BMI, systolic blood pressure, diastolic blood pressure, hypertension medication use, lipid-lowering medication use, insulin use, and oral hypoglycemic medication use. High TG defined as TG > 160 mg/dL; low HDL defined as ≤40 mg/dL (men) or ≤50 mg/dL (women). Test for interaction: *p* = 0.94.

In a secondary analysis (Fig. 1), we assessed hazard ratios and 95% confidence intervals for MACE stratified by Tg/HDL-C phenotypes and LDL-C levels together. When compared to the loTg/hHDL plus LDL-C < 100 group (i.e., the lowest risk group for CVD events), hazard ratios increased ∼2.0 fold in both the intermediate and hTg/LoHDL phenotypic groups once LDL was >100 mg/dl, with no significant evidence of synergy (*p* = 0.94).

## 4 Discussion

The present study utilized lipid phenotyping to risk stratify CHS participants with type 2 diabetes. LDL cholesterol has been demonstrated in numerous studies to be a risk factor for CVD outcomes in type 2 diabetes [23]. Our data demonstrate significant differences in CVD outcomes in diabetic subjects based on their lipid phenotype. Importantly, the hLDL phenotype did not clearly confer the highest risk, but rather the hTg/loHDL phenotype appeared at least as strong. Analysis of lipid values as continuous variables similarly demonstrated strong associations with high triglycerides as well as non-HDL cholesterol and remnant cholesterol that were at least as strong as LDL-C. Thus, a high triglyceride phenotype clearly marks high cardiovascular risk among older diabetic adults.

With that said, it may be that not all (or any) of these risk factors are targetable disease modifiers. The rationale for lipid phenotyping is based on the underlying molecular genetics and biology of various known dyslipidemias. Lipid phenotyping is also an attempt to break away from a reductionist view of CVD risk in diabetic individuals that rely primarily on LDL cholesterol. Patients with type 2 diabetes typically receive statin-drugs as a standard of care for cardiovascular disease (CVD) risk prevention to lower LDL cholesterol. Such individuals with or without lipid abnormalities have a 3–8 times increased risk for CVD compared to individuals without diabetes [24].

Manson and Haffner used the Nurses’ Health Study (NHS) with > 110,000 study subjects followed for 20 years to show that female subjects with type 2 diabetes had significantly increased risk of CVD [25]. Compared to women who were non-diabetic throughout the NHS, those with type 2 diabetes at baseline entry into the study had an adjusted relative CVD risk of 5.02. Intermediate risks were found among participants who entered the study prior to developing diabetes. These subjects, once they developed diabetes, were risk stratified based on the time-period before diabetes onset or after diabetes onset. Those individuals had higher CVD risk after the onset of diabetes than before onset during the study period. Other studies showed similar data that patients with type 2 diabetes have increased risk of developing CVD over ∼10 to 20 years [26]. These well-conducted studies led to the present standard of aggressive lipid lowering with statin drugs to a target LDL cholesterol of <70 mg/dl in all patients with type 2 diabetes and has remained unchanged for the past 20 years.

Of note, new therapeutic options for patient with type 2 diabetes including GLP-1 receptor agonists and SLGT-2 inhibitors appear to confer cardiovascular risk protection through diverse mechanisms, but these may include altering lipid profiles [27,28]. How much of their benefit relates to each of these diverse mechanisms remains to be determined.

The conceptual framework for statin treatment in all patients with type 2 diabetes arose from a misuse of the transitive law. First, patients with type 2 diabetes are known to have equal CVD risk as a non-diabetic individual with a first MI. Second, all patients with a first MI are treated with a statin to reduce CVD risk. Therefore, all diabetic subjects need to be treated with statins since their CVD risk is equal to first MI subjects. While useful for the past 20–30 years, this approach can be improved by combining newer insight into the molecular biology of lipid disorders, common lipid phenotypes, and clinical outcomes data.

The major present day CVD risk calculator is based on 2013 American College of Cardiology/American Heart Association guidelines updated in November 2017. This calculator includes age in years, sex (M or F), race (Black or other) total cholesterol, HDL cholesterol, systolic and diastolic BP, treatment for hypertension (yes or no), diabetes (yes or no) and smoker (yes or no). The risk score is tied to guidelines for treatment with statin and aspirin or not. Fasting triglyceride level is not considered in this calculation and neither (by definition) is lipid phenotype. Of note, the ADVANCE (Action in Diabetes and Vascular Disease) Study revealed that both the Framingham and UKPDS risk equations are unreliable, with overestimation of CVD risk by ∼150–250% [29].

We previously published data showing a plot of CVD relative risk reduction (RRR) in six major statin studies versus fasting triglyceride level (represented as the mean of the treated study population). Linear regression analysis revealed a line with coefficient of variance (r^2^) = 0.59. For every 10 mg/dl increase in mean fasting triglyceride level the statin-induced CVD RRR was decreased by ∼2.5% [30]. This led us to the hypothesis that fasting triglyceride level per se plays a significant role in CVD risk reduction with statin use. Moreover, we speculated that fasting triglyceride and HDL cholesterol could be combined into a specific lipid phenotype since the metabolic pathways from VLDL to small dense LDL and small dense HDL are interconnected when insulin resistance is present [31].

Prior work from the Cardiovascular Health Study (CHS) reveals strong clues that support our lipid phenotype hypothesis. Cluster analysis of six cardiometabolic groups demonstrated those with higher levels of insulin resistance had a CVD hazard ratio of 2.26 compared to those non-insulin resistant groups [32]. This suggested that insulin resistance, which includes the high triglyceride plus low HDL phenotype, might be useful in a formalized risk calculator to better define CVD risk groups. Additional evidence of triglyceride-mediated CVD risk comes from analysis of polymorphisms of the APOC5 promoter in several large patient populations. Here, the research group analyzed the allelic frequency of −1131T>C in the study population. They determined that for every C allele inherited, mean triglyceride level was 16% higher and was associated with higher VLDL particle concentration and small dense HDL (a known atherogenic particle). CVD risk increased with C-allele frequency in this study [33]. Indeed, Nanna and colleagues showed that pooled cohort risk equations, in general, did poorly at predicting 5-year ASCVD risk with a noted overestimation of risk in the highest risk groups [34]. It is therefore possible that additional biomarkers such as lipoprotein particle size and density might offer useful information on CVD risk assessment when integrated with lipid phenotyping [35].

We see additional evidence from genetic studies supporting the lipid phenotype hypothesis. Exome-wide association studies have implicated triglyceride lowering alleles that increase lipoprotein lipase (LPL) activity (e.g., LPL and ANGPTL4). LPL activity increased peripheral lipolysis of triglycerides and led to lower CVD and type 2 diabetes risk [36]. The effect of triglyceride lowering was further established through analysis of loss of function gene mutations in the human APOC3 gene (the apoprotein that inhibits lipoprotein lipase activity). These rare individuals with loss of function mutations had triglyceride levels ∼40% lower than wild type APOC3 and ∼40% decreased risk for CVD [13]. Moreover, Mendelian randomization studies of SNP's involved in plasma triglyceride levels showed strong association for CVD with odds ratios ∼1.6 [37]. Therefore, human evidence from phenotype to genotype appears to support the hypothesis that fasting triglyceride (with or without concurrent low HDL) plays a role in cardiovascular risk that is not accounted for by today's CVD risk calculators.

Our study reveals that diabetic subjects with the hTg/loHDL phenotype had similar relative risks for HF, MI, and MACE and significantly higher risk for stroke compared to those individuals with the hLDL phenotype. This finding demonstrates that LDL-C alone should not be the sole target for strategies to reduce CVD risk in diabetic subjects. Furthermore, lipid phenotyping provides a simple approach to better categorizing different risk profiles in individuals with type 2 diabetes.

Indeed, both the Reduce-It and Jelis studies demonstrated that a focus on LDL cholesterol is not enough. In the Reduce-It Study, participants were stratified to a control group that received simvastatin alone or a treatment group that received equal dose simvastatin plus eicosapentanoic acid (icosapent ethyl) [38]. Approximately 60 percent of study subjects had type 2 diabetes and the median triglyceride level was 216.5 mg/dl. The addition of icosapent ethyl conferred an additional 25 percent CVD risk reduction compared to the group treated with statin only. In the Jelis study, EPA addition to statin treatment lowered CVD events by 53 percent compared to a statin-only control group [39].

Strengths of this study relate to the prospective study design of CHS in addition to its longevity and data abundance. As an ongoing effort for the past 30 years, nearly all CHS participants have been followed to death. Therefore, the data repository has excellent CVD outcomes and demographic risk factors. Moreover, the low use of statin drugs at baseline allowed us to obtain pristine lipid profiles in study participants. Most other studies with CVD outcomes have much higher rates of prior statin “contamination” that could significantly affect lipid phenotype assignments. Assignments of study subjects having diabetes were determined by baseline glucose and use of glucose lowering agents.

There are several limitations to our study. This is a relatively small study with participants recruited in 1989–1993. Dietary habits, exercise, and life-stressors have changed over the ensuing three decades; therefore, it is possible that other confounding factors are not fully accounted for in this observational study. The ethnic composition of CHS is primarily White and Black subjects, with only a small percent of other ethnicities represented. Therefore, we are not able to generalize our phenotyping results. Diabetes duration was not ascertained at baseline. Hence, the impact of disease severity on CVD outcome could not be accounted for in the analysis. Finally, we cannot extrapolate our data to treatment recommendations for those diabetic individuals without the hLDL phenotype until randomized clinical trials validate or refute our proposition.

Future studies are needed to determine whether statin therapy is the optimal treatment for the hTg/loHDL group or whether direct triglyceride lowering agents are more appropriate. This question was partially addressed in the ACCORD lipid study (simvastatin versus simvastatin plus fenofibrate) where fenofibrate was found not to improve CVD outcomes in type 2 diabetic subjects on statin therapy [40]. Unfortunately, this study does not provide us with incontrovertible proof of their hypothesis because approximately sixty percent of ACCORD lipid study participants had prior exposure to statins and mean fasting triglyceride was only ∼162 mg/dl for all subjects. This is not an optimal profile for treatment of the hTg/loHDL phenotype. In CHS, lipid lowering therapy (primarily statins) prior to enrollment in the hTg/loHDL phenotype ranged from 5 to 10 percent, with mean fasting triglyceride ∼236 mg/dl. Indeed, a post-hoc subgroup analysis of ACCORD lipid data demonstrated that significant CVD risk reduction occurred in ACCORD lipid study participants with fasting triglyceride levels ≥ 200 mg/dl [41]. However, the more recent PROMINENT CV study using pemafibrate to lower triglycerides in an appropriate study group with T2DM (mean baseline fasting triglyceride level was 271 mg/dl) did not show CVD risk reduction [4]. This study moves the needle away from using triglyceride level per se as a biological target for CVD risk reduction, at least until ApoC3-targeted strategies have been vetted.

In conclusion, we demonstrate that in CHS participants with type 2 diabetes CVD risk can be apportioned not only to elevated LDL cholesterol, but to diverse lipid phenotypes that include high triglycerides and low HDL cholesterol. This data should spur efforts to revamp cardiovascular risk stratification in subjects with type 2 diabetes since older methods do not account for a broad spectrum of lipid phenotypes.

## Disclosures

DB has no conflicts of interest. MLB has no conflicts of interest, JMG has no conflicts of interest. ML has no conflicts of interest. DS has no conflicts of interest. KM has no conflicts of interest. The content is solely the responsibility of the authors and does not necessarily represent the official views of the National Institutes of Health.

## Data sharing

CHS data are available to qualified investigators following study policies and procedures https://chs-nhlbi.org/CHS_DistribPolicy or through the BioLINCC data repository: https://biolincc.nhlbi.nih.gov/home/

## Funding sources

KM has funding from National Institute on Aging K24AG065525. This research was supported by contracts HHSN268201200036C, HHSN268200800007C, HHSN268201800001C, N01HC55222, N01HC85079, N01HC85080, N01HC85081, N01HC85082, N01HC85083, N01HC85086, 75N92021D00006, and grants U01HL080295 and U01HL130114 from the National Heart, Lung, and Blood Institute (NHLBI), with additional contribution from the National Institute of Neurological Disorders and Stroke (NINDS). Additional support was provided by R01AG023629 from the National Institute on Aging (NIA). A full list of principal CHS investigators and institutions can be found at CHS-NHLBI.org."

## CRediT authorship contribution statement

**David Bleich:** Writing – review & editing, Writing – original draft, Supervision, Conceptualization. **Mary L. Biggs:** Methodology, Formal analysis, Data curation. **Julius M. Gardin:** Writing – review & editing, Supervision. **Mary Lyles:** Investigation, Data curation. **David S. Siscovick:** Writing – review & editing, Conceptualization. **Kenneth J. Mukamal:** .

## Declaration of competing interest

The authors declare the following financial interests/personal relationships which may be considered as potential competing interests:

Ken Mukamal, MD reports financial support was provided by National Institute on Aging. If there are other authors, they declare that they have no known competing financial interests or personal relationships that could have appeared to influence the work reported in this paper.

## Appendix Supplementary materials

Image, application 1
