# Supplementary material for: Phenotyping lipid profiles in type 2 diabetes: Risk association and outcomes from the Cardiovascular Health Study
Source: Am J Prev Cardiol. 2024 Aug 26;19:100725. doi: 10.1016/j.ajpc.2024.100725 (PMC11402907; doi:10.1016/j.ajpc.2024.100725)
Supplement: Supplementary file 1 [file mmc1.docx]

**Phenotyping Lipid Profiles in Type 2 Diabetes: Risk Association and Outcomes from the**

**Cardiovascular Health Study.**

David Bleich^1^, Mary L. Biggs^2^, Julius M. Gardin^1^, Mary Lyles^3^, David Siscovick^4^ and Kenneth Mukamal^5^

^1^Rutgers New Jersey Medical School, Newark, NJ, ^2^University of Washington School of Medicine, Seattle, WA, ^3^Wake Forest School of Medicine, Winston-Salem, NC, ^4^New York Academy of Medicine, New York, NY, ^5^Harvard Medical School, Boston, MA.

**Supplemental Tables**

Supplemental Table 1. Characteristic of CHS participants with diabetes at baseline, by LDL phenotype

|  | LDL (mg/dL) | | |
| --- | --- | --- | --- |
|  | <100  (n=221) | >=100-<130  (n=279) | >=130  (n=366) |
| Age (years) | 73.8 ± 6.1 | 73.0 ± 5.2 | 72.3 ± 5.2 |
| Male | 62.9% | 53.8% | 38.3% |
| Black race | 16.7% | 28.0% | 24.9% |
| Field center |  |  |  |
| Bowman Gray | 28.5% | 22.6% | 25.7% |
| Davis | 18.1% | 21.5% | 21.9% |
| Hopkins | 25.8% | 23.7% | 24.3% |
| Pittsburgh | 27.6% | 32.3% | 28.1% |
| Educational attainment |  |  |  |
| <HS | 38.9% | 34.8% | 36.9% |
| HS | 22.2% | 27.6% | 29.8% |
| >HS | 38.9% | 37.6% | 33.3% |
| Body mass index (kg/m2) | 138.6 ± 22.3 | 140.4 ± 20.4 | 141.5 ± 22.1 |
| Diastolic BP (mmHg) | 70.0 ± 12.1 | 70.2 ± 11.9 | 71.6 ± 11.7 |
| Systolic BP (mmHg) | 80.2 ± 15.5 | 115.3 ± 8.7 | 160.8 ± 26.3 |
| LDL (mg/dl) | 138.6 ± 22.3 | 140.4 ± 20.4 | 141.5 ± 22.1 |
| HDL (mg/dl) | 45.9 ± 15.0 | 48.2 ± 13.3 | 48.7 ± 11.3 |
| Triglyceride (mg/dl) | 167.7 ± 91.9 | 146.5 ± 60.3 | 163.0 ± 64.6 |
| Fasting glucose (mg/dl) | 167.0 ± 65.4 | 168.9 ± 51.1 | 173.4 ± 63.5 |
| Any anti-hypertensive medication | 67.4% | 63.4% | 61.7% |
| Any lipid-lowering medication | 5.0% | 7.2% | 9.6% |
| Oral hypoglycemic agents | 42.1% | 39.8% | 39.6% |
| Insulins | 17.6% | 12.5% | 13.9% |
| CHD | 29.9% | 27.6% | 26.8% |
| CHF | 11.3% | 8.6% | 7.1% |
| Stroke | 10.0% | 5.4% | 6.3% |
| No. alcoholic beverages/wk | 1.9 ± 5.7 | 1.6 ± 5.0 | 1.2 ± 4.0 |
| Physical activity (kcal/wk) | 1777.8 ± 2234.9 | 1383.8 ± 1845.0 | 1399.9 ± 2033.2 |
| Smoking status |  |  |  |
| Never smoked | 47.5% | 44.8% | 46.7% |
| Former smoker | 46.2% | 42.3% | 43.4% |
| Current smoker | 6.3% | 12.9% | 9.8% |

Figures shown are mean ± SD for continuous measures and percentages for categorical measures.

Supplemental Table 2. Crude incidence of events among CHS participants with diabetes at baseline, by LDL phenotype

|  | **LDL (mg/dL)** | | | | | |
| --- | --- | --- | --- | --- | --- | --- |
| **Event** | **<100** | | **>=100-<130** | | **>=130** | |
|  | No. | Incidence per 1,000 | No. | Incidence per 1,000 | No. | Incidence per 1,000 |
| All-cause mortality | 216 | 96.1 | 270 | 89.7 | 348 | 88.4 |
| CVD mortality | 86 | 38.2 | 133 | 44.2 | 177 | 45.0 |
| MI | 37 | 21.0 | 62 | 25.4 | 82 | 25.0 |
| CHF | 82 | 45.9 | 127 | 55.4 | 151 | 46.3 |
| Stroke | 43 | 22.1 | 52 | 19.4 | 92 | 26.9 |
| MI, Stroke, HF, CVD mortality | 104 | 76.3 | 154 | 81.5 | 208 | 71.1 |

Supplemental Table 3. Crude incidence of events among CHS participants with diabetes at baseline, by TG/HDL phenotype

|  | **TG/HDL*** | | | | | |
| --- | --- | --- | --- | --- | --- | --- |
| **Event** | **Low TG/High HDL** | | **Indeterminate** | | **High TG/Low HDL** | |
|  | No. | Incidence per 1,000 | No. | Incidence per 1,000 | No. | Incidence per 1,000 |
| All-cause mortality | 316 | 85.7 | 277 | 94.3 | 241 | 93.8 |
| CVD mortality | 138 | 37.4 | 132 | 44.9 | 126 | 49.0 |
| MI | 68 | 21.6 | 59 | 25.0 | 54 | 27.4 |
| CHF | 128 | 42.2 | 122 | 51.7 | 110 | 56.4 |
| Stroke | 52 | 15.7 | 74 | 29.3 | 61 | 27.4 |
| MI, Stroke, HF, CVD mortality | 164 | 66.1 | 170 | 94.1 | 132 | 90.4 |

*High TG defined as TG > 160 mg/dL; low HDL defined as ≤40 mg/dL (men) or ≤50 mg/dL (women)
